# Clinicopathological Predictors of Recurrence in Uterine Sarcomas—A Narrative Review

**DOI:** 10.3390/jcm14144883

**Published:** 2025-07-09

**Authors:** Emmanuel N. Kontomanolis, Ioakeim Sapantzoglou, Konstantinos Nikolettos, Evangelia Kontogeorgi, Vasiliki Lampraki, Dimitrios Papageorgiou, Paraskevas Perros, Zacharias Fasoulakis, Aristotelis-Marios Koulakmanidis, Maria-Anastasia Daskalaki, Vasilios Pergialiotis, Panagiotis Antsaklis, Marianna Theodora, George Daskalakis

**Affiliations:** 1 Department of Obstetrics and Gynecology, Democritus University, 67100 Alexandroupolis, Greece; mek-2@otenet.gr; 2 1st Department of Obstetrics and Gynecology, Alexandra Hospital, National and Kapodistrian University of Athens, Vasilissis Sofias 80 Aven., 11528 Athens, Greece; evangelia-kont@hotmail.com (E.K.); vaslampraki93@yahoo.com (V.L.); paris_per@yahoo.gr (P.P.); hzaxos@gmail.com (Z.F.); aristoteliskoulak@gmail.com (A.-M.K.); anastasia.daskalaki00@gmail.com (M.-A.D.); pergialiotis@yahoo.com (V.P.); panosant@gmail.com (P.A.); martheodr@med.uoa.gr (M.T.); gdaskalakis@yahoo.com (G.D.); 3 Department of Gynecological Oncology, Maidstone and Turnbridge Wells, NHS Trust, Maidstone TN2 4QJ, UK; k.nikolettos@yahoo.gr; 4 Department of Gynecology, Athens Naval and Veterans Hospital, 11521 Athens, Greece; dimitris_papageorgiou@outlook.com

**Keywords:** uterine sarcomas, recurrence, leiomyosarcoma, carcinosarcoma, endometrial stromal sarcoma, predictors

## Abstract

**Background:** Sarcomas are a rare and biologically diverse group of malignant tumors that originate from mesenchymal tissues. They are characterized by a broad range of histopathological subtypes, varying clinical courses, and differing responses to treatment. This study seeks to clarify the clinicopathological and molecular predictors of recurrence in leiomyosarcomas, carcinosarcomas, and endometrial stromal sarcomas to enhance our understanding, thereby improving clinical knowledge, consultation practices, and the overall benefit for patients. **Methods**: A literature search was conducted utilizing PubMed/MEDLINE, Embase, Cochrane Library, and Scopus to execute a comprehensive structured narrative review of articles published up to 31 March 2025. **Results**: We summarize existing evidence on the clinical, histological, and molecular predictors of recurrence and poor prognosis for leiomyosarcomas, carcinosarcomas, and endometrial stromal sarcomas. While the stage, grade, tumor size, and novel molecular biomarkers are crucial high-risk parameters that have been associated with recurrence, existing data demonstrate contradictory results, indicating the need for further research. **Conclusions**: Recent advancements in next-generation sequencing have facilitated the identification of women at increased risk of recurrence, poor disease-free survival, and overall adverse prognosis. Stratifying this risk requires a comprehensive understanding of the clinical, histological, and molecular risk factors involved. Understanding these underlying factors is essential for effectively addressing the initial consultation, guiding management, and—considering the novel treatment modalities—individualizing the care provided to the affected women.

## 1. Introduction

Sarcomas represent a rare and biologically heterogeneous group of malignant neoplasms originating from mesenchymal tissues, distinguished by a wide spectrum of histopathological subtypes, variable clinical trajectories, and diverse therapeutic responses. Sarcomas account for approximately 1% of all malignancies of the female genital tract and represent about 3–7% of uterine cancers [1,2].

Uterine sarcomas are subclassified into homologous and heterologous types. Homologous sarcomas arise from mesenchymal elements normally present in the uterus, such as uterine leiomyosarcoma (u-LMS, representing 30% of uterine sarcomas) and endometrial stromal sarcoma (ESS) (representing 15% of uterine sarcomas). Heterologous sarcomas originate from cell types not typically found in the uterus, including rhabdomyosarcoma, liposarcoma, and carcinosarcoma. Carcinosarcomas (also referred to as “malignant mixed Müllerian tumors”) account for 50% of uterine sarcomas and, although previously categorized as a subtype of uterine sarcoma owing to their biphasic histology, they have been recently reclassified by the World Health Organization as high-grade endometrial carcinoma with sarcomatoid differentiation [3,4].

Uterine sarcomas are associated with a significantly poorer prognosis and exhibit more aggressive clinical behavior compared to endometrial carcinomas. Five-year survival rates range from 30% to 48%, and relapse rates approach 60%, with 42% of relapses occurring outside the pelvis [5]. This is attributed not only to their intrinsic biological aggressiveness but also to the absence of specific early symptoms, limitations in diagnostic modalities, and a lack of well-established, standardized treatment protocols. Prognosis is significantly influenced by the histopathological subtype and the extent to which optimal multimodal therapy can be achieved [6].

The present study aims to elucidate the underlying clinicopathological and molecular predictors of recurrence for leiomyosarcoma, carcinosarcoma, and endometrial stromal sarcomas that have been investigated in the existing literature. We do so to enrich our understanding of their nature, aiming to improve clinical knowledge, consultation practices, and the overall survival of affected patients.

## 2. Materials and Methods

**Figure 1:**
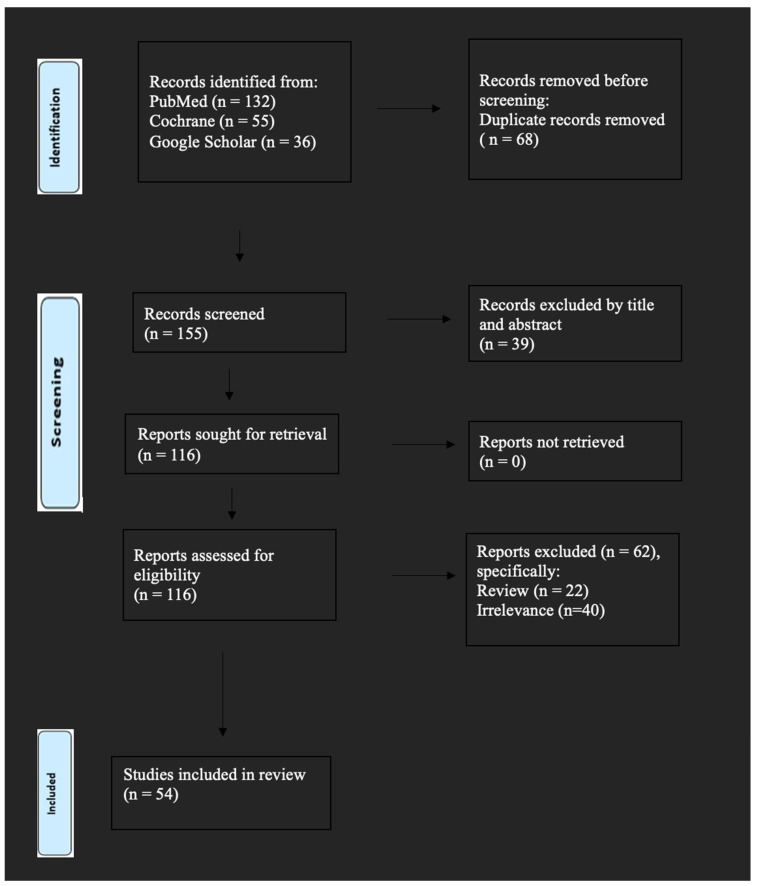
Search strategy for the included studies.

A literature search was conducted utilizing PubMed/MEDLINE, Embase, Cochrane Library, and Scopus to execute a comprehensive structured narrative review of articles published up to 31 March 2025, employing the following search terms: “uterine sarcomas” AND “recurrence”, OR “leiomyosarcoma”, “recurrence” OR “carcinosarcoma”, “recurrence” OR “endometrial stromal sarcoma”, “recurrence”. The chosen articles were mandated to be original works composed in English. Included studies contained systematic reviews, original studies, and case reports/series. Commentaries and news pieces were omitted. The studies underwent independent assessment by the author I.S. The references of these papers were examined for any potentially overlooked studies. All publications found in prior systematic reviews were incorporated. Our search identified 223 potentially relevant studies, but 169 were excluded after reviewing the titles and abstracts and after the exclusion of non-relevant articles, case reports, opinion letters, reviews, and letters to the editor. Overall, 54 studies were included in the present systematic review. The search strategy is briefly presented in Figure 1. The methodological characteristics and summarized results of the included studies for uterine leiomyosarcomas, carcinosarcomas, and endometrial stromal sarcoma are depicted in Table 1, Table 2, and Table 3, respectively.

## 3. Results

### 3.1. Uterine Leiomyosarcoma (u-LMS)

#### 3.1.1. Demographic Characteristics

The age at initial u-LMS diagnosis has been repeatedly reported as a prognostic factor of overall survival (OS) and recurrence-free survival (RFS). In their retrospective analysis of 94 patients with different histologic types of uterine sarcoma, Denschlag et al. demonstrated that in both univariate and multivariate analysis, OS was significantly associated with patient age [7]. Similarly, in a cohort of 52 patients with mesenchymal uterine tumors, including 20 cases of u-LMS, D’Angelo et al. found that patient age was a significant contributor to the prognosis according to a univariate analysis; however, this correlation was not confirmed after a multivariate analysis [8]. Furthermore, these findings have been confirmed in larger study groups by Kapp et al. and Tirumani et al., who investigated prognostic factors and survival in cohorts including 1396 and 113 patients with uterine sarcomas, respectively, which was mostly attributed to the presentation of higher-grade u-LMS in older patients [9,10].

In accordance with the above, several studies have also implicated menopausal status in the poor prognosis and increased recurrence rates of u-LMS. A 1986 study investigating prognostic factors and several treatment modalities in 209 patients—81 with confirmed LMS and a 2-year recurrence rate of 23%—revealed that the strongest prognostic marker of OS in their cohort was menopausal status [11]. Similar results have been demonstrated in a more recent study by Wang et al., in which univariate analysis associated both menopausal status and age above 50 years old with poor outcomes; however, after a multivariate analysis, only menopause retained its statistical significance [12]. 

#### 3.1.2. Stage, Grade, and Tumor Size

The tumor stage is the paramount prognostic determinant. Historically, uterine sarcomas were classified according to a staging method introduced in 1988 for endometrial cancer. However, this has not demonstrated adequacy and, as such, in 2009, a novel FIGO staging method was established for uterine sarcomas. This updated staging system comprises two divisions: one for leiomyosarcoma and endometrial stromal sarcoma (ESS) and another for adenosarcoma. Carcinosarcoma is currently staged according to the endometrial carcinoma staging system [59]. Various studies have shown inconsistency in the correlation between survival and factors such as clinical stage, grade, and tumor size, with these factors being the most commonly investigated parameters in terms of recurrence and OS [60]. Several older studies have demonstrated that the FIGO tumor stage is a significant contributor to OS and recurrence rates [8,13,14]. Specifically, D’Angelo et al. revealed that stage was the only parameter that remained statistically significant after multivariate analysis [8]. However, parameters such as tumor size and mitotic count did not demonstrate any significant correlation with survival, findings that were inconsistent with the results of a previous study by Abeler et al. involving 245 leiomyosarcomas confined to the uterus. They identified tumor size and the mitotic index as significant prognostic factors, eventually facilitating stratification of the patients into three distinct risk groups, demonstrating notable differences in prognosis [15]. However, in their next study which included a larger study group, the same authors demonstrated that a tumor size greater than 10 cm (with all stages included) demonstrated prognostic significance in both univariate and multivariate analyses [16]. More recent studies have produced contradictory results regarding the association of tumor stage with OS and recurrence, but most of them agree that tumor size constitutes an independent risk factor both for OS and progression-free survival (PFS) [6,10,12,17,18]. Specifically, Dermawan et al. [18] and Wang et al. [6] reviewed data from 177 and 63 patients with u-LMS of any stage, demonstrating that a tumor size above 10 cm and 7.5 cm, respectively, is correlated with an unfavorable outcome in terms of OS and recurrence, and its prognostic significance seems to remain even in uterine-confined disease (FIGO Stage 1) [17]. The u-LMS grade has also been investigated, but only two of the retrieved studies revealed an association between a documented high-grade tumor and poor prognosis; however, the authors acknowledged a number of limitations in their studies and a lack of generalizable results [9,14].

#### 3.1.3. Mitotic Index/Count

The mitotic index quantifies the proportion of cells undergoing division observed in a high-power field under a microscope. A combination of 15 mitotic figures per 10 high-power fields (MF/10 HPF), along with hypercellularity and severe nuclear atypia, typically characterizes malignant smooth muscle tumors of the uterus [2]. Several published studies have associated the detection of a high mitotic count with a worse prognosis, albeit using different cut-offs [15,16,17]. D’Angelo et al. stratified patients into two categories demonstrating different prognoses, with a mitotic index of ≥20 MF/10 HPF predicting adverse prognosis, while Chen et al. used a cut-off of >10/10HPF as an independent prognostic factor. Another study including 349 patients with either intra-abdominal or distant metastatic disease revealed that a mitotic count >10M/10 HPF was the only parameter that carried a statistically significant risk of poor prognosis [19]. The significance of the mitotic index was further highlighted through its implementation—along with six other clinicopathologic features—in a u-LMS-specific nomogram that was created to more precisely stratify patient groups regarding their post-surgery prognosis [61]. This model was both internally and externally validated [62], demonstrating a prediction accuracy very close to actual outcomes in terms of overall survival.

#### 3.1.4. Molecular Biomarkers

Several molecular markers have been investigated to assess their contributions to the prediction of recurrence and OS in patients with u-LMS [8,13,16,17,18,19]. ki67 and p53 were thoroughly explored by Zhai et al. [63], who reported a statistically increased number of ki67 positive cells and abnormally high expression of the oncogene p53 tumor-suppressor gene in cases of u-LMS compared with cellular and usual leiomyoma, as well as with tumors of uncertain malignant potential. The authors demonstrated that ki67 was useful in differentiating these entities from each other. More recent studies have demonstrated that the strong expression of ki-67 and p53 is associated with recurrence and poor OS in long-term follow-up [8,17]. For example, in their 2009 study, D’Angelo et al. revealed that negative u-LMS tumors or those expressing low levels of Ki-67, p53, p16, and Twist were associated with a more favorable outcome. The same authors conducted a follow-up study 2 years later, concluding that tumors measuring 10 cm or more in diameter, exhibiting 20 or more mitotic figures per 10 high-power fields, demonstrating 10% or greater immunoreactive nuclei for Ki67, and testing negative for Bcl-2 were associated with a poorer prognosis [8,16]. Conversely, an investigation of bcl-2 (a biomarker correlated with cellular apoptosis) produced contradictory results. Lusby et al. concluded that increased bcl-2 levels may predict longer disease-specific survival, while other authors have associated its presence and expression with an adverse outcome [8,16,19]. b-catenin (a contributing factor to the Wnt signaling pathway) has also been investigated, with the overexpression of its nuclear subtype being associated with both the extension of malignancy outside the uterus at initial diagnosis and intraperitoneal recurrence [19,20]. Wilms tumor gene 1 (WT1) has also been identified as an independent prognostic marker for OS [13], and a recent study has demonstrated that dystrophin-negative u-LMS was associated with worse overall survival than dystrophin-positive u-LMS malignancies [21]. Recently, Dermawan presented a genomic model of stratification patients with u-LMS that demonstrated a significantly increased risk of poor progression-free survival and disease-specific survival when TP53 mutation and chr20q amplification/ATRX mutations were present at the same time. This study group concluded that such a stratification outperforms traditional clinicopathologic models in predicting clinical outcomes [18].

The characteristics and results of the included studies are summarized in Table 1.

### 3.2. Uterine Carcinosarcoma (UCS)

Uterine carcinosarcomas—also known as malignant mixed Müllerian tumors—are high-grade, endometrial-originated neoplasms that comprise about 5% of endometrial cancers, characterized by poor prognosis [64,65].

Although uterine carcinosarcoma was historically categorized as a type of sarcoma, advances in molecular and genetic research have shown that the sarcomatous element of carcinosarcomas arises through trans-differentiation from the epithelial (carcinomatous) component. Consequently, UCS is now widely regarded as a form of metaplastic endometrial carcinoma [22,23,24,66].

#### 3.2.1. Demographic Characteristics

Uterine carcinosarcoma is typically considered an elderly disease, with peak incidence reported in the 70–79-year age group. However, Matsuzaki S. et al. reported a significant increase in the age-adjusted incidence rate of UCS, rising from 1.0 to 1.4 per 100,000 between 2000 and 2016 [67]. During the same period, the incidence rate increased by 1.7% annually (95% CI, 1.2–2.2) [25,67]. Notably, women aged 60–69 years exhibited the largest interval increase in incidence, with an annual percent change (APC) of 2.7% (95% CI, 1.9–3.4, *p* < 0.001), followed by those aged 70–79 years (APC 2.0%, 95% CI, 1.2–2.9, *p* = 0.001) and 50–59 years (APC 1.2%, 95% CI, 0.5–2.0, *p* = 0.002). This trend reflects a decrease in the average age of diagnosis, which has shifted from 71.7 years to 67.0 years between 1989 and 2013 [67].

An elevated risk of uterine carcinosarcoma has been reported among black women and those with obesity, aligning with known risk factors for endometrial malignancies [26,27,28]. According to Matsuzaki et al., black women exhibit a disproportionately higher incidence of uterine carcinosarcoma compared to other racial and ethnic groups, with an age-adjusted rate of 2.9 per 100,000, in contrast to 0.8–1.2 per 100,000 among other populations. Of note, the greatest interval increase in UCS incidence from 2000 to 2016 was observed among Hispanic women (annual percent change [APC] 2.7; 95% CI, 1.7–3.6; *p* < 0.001), followed by black (APC 2.3; 95% CI, 1.4–3.3; *p* < 0.001) and white women (APC 1.1; 95% CI, 0.5–1.7; *p* = 0.002) [67].

#### 3.2.2. Stage, Grade, and Tumor Size

Uterine carcinosarcoma is associated with a notably poor prognosis across all stages. According to SEER, while approximately 43.9% of uterine carcinosarcoma cases are diagnosed at stage I, a substantial proportion present with advanced-stage disease, with 8.7% at stage II, 22.9% at stage III, and 24.4% at stage IV [68].

As reported in recent reviews, even patients with stage I disease experience limited survival, with a 5-year overall survival rate of 54.8%. Survival decreases significantly with disease progression, with corresponding 5-year OS rates of 36.9% for stage II, 24.9% for stage III, and only 9.2% for stage IV (*p* < 0.001). Median OS similarly declines from 78 months in stage I to 30, 19, and 8 months in stages II, III, and IV, respectively. These data underscore the strong prognostic value of disease stage in determining survival outcomes in patients with uterine carcinosarcoma [3,67].

Several additional clinicopathological parameters have been shown to negatively influence 3-year overall survival in uterine carcinosarcoma. These include a primary tumor diameter ≥5 cm (hazard ratio [HR] 2.23; 95% confidence interval [CI] 1.32–3.77; *p* = 0.003), deep myometrial invasion (HR 2.82; 95% CI 1.77–4.48; *p* = 0.001), lymphovascular space invasion (LVSI) (HR 2.11; 95% CI 1.26–3.52; *p* = 0.005), rhabdomyoblastic differentiation of the sarcomatous component (HR 2.58; 95% CI 1.30–7.35; *p* = 0.046), and the presence of residual tumor >1 cm after surgery (HR 1.75; 95% CI 1.07–2.84; *p* = 0.0245) [29,69]. Furthermore, a rising trend in lymph node metastasis has been noted over time, with nodal involvement observed in nearly 25% of UCS cases as of 2016 [29,68]. 

The carcinomatous component of uterine carcinosarcomas—particularly when high-grade—has been identified in multiple studies as an adverse prognostic factor. This may be attributed to the intrinsically aggressive behavior of the epithelial element, which is more frequently associated with metastatic dissemination and lymphovascular invasion than the mesenchymal component [30,31,32]. Nordal et al. demonstrated that serous and clear-cell histologic subtypes within the carcinomatous component are associated with an adverse prognosis. In contrast, the histologic grade of differentiation of the carcinomatous component did not appear to influence clinical outcomes [25]. Kim et al. investigated the prognostic impact of the heterologous element in gynecologic carcinosarcomas. In their meta-analysis, including uterine and ovarian carcinosarcomas, the presence of heterologous components was significantly associated with decreased overall survival, but no significant correlation was found with pooled RFS or disease-free survival (DFS), further supported by a subgroup analysis [32]. 

#### 3.2.3. Molecular Biomarkers

Multiple molecular classifications have been explored for their potential prognostic relevance in UCS. The Cancer Genome Atlas (TCGA) has proposed four molecular subgroups: the POLE-ultra mutated (POLEmut) subtype, associated with favorable prognosis; the microsatellite instability/mismatch repair-deficient (MSI/MMRd) group and the no specific molecular profile (NSMP) group, both associated with intermediate prognosis; and the TP53-mutant/p53-abnormal (TP53mut/p53abn) subtype, which is linked to poor clinical outcomes [22,33,34,35,36,37,38,70]. According to Tavaglino et al., patients with NSMP and TP53mut/p53abn UCS demonstrate inferior progression-free survival (PFS) compared to those with MSI/MMRd tumors (HR of 0.19 (95% confidence interval [CI] 0.08–0.46; *p* < 0.001)). However, overall survival was comparable between the MSI/MMRd, NSMP, and TP53mut/p53abn subgroups (HR values of 0.91 (95% CI 0.44–1.87; *p* = 0.788) and 1.51 (95% CI 0.76–2.99; *p* = 0.240)). Among patients with POLE-mutated tumors, no cases of disease progression or death were observed during the follow-up period, indicating an excellent prognosis in terms of both progression-free and overall survival [71].

The epithelial–mesenchymal transition (EMT) is a biological process in which epithelial cells lose their apical–basal polarity and intercellular adhesion, acquiring mesenchymal properties such as enhanced motility, invasiveness, and resistance to apoptosis [72,73]. EMT is significantly involved in the sarcomatous dedifferentiation observed in uterine carcinosarcomas [39]. Among the commonly altered genes in these tumors, such as TP53, PIK3CA, FBXW7, PTEN, and ARID1A, the tumor suppressor FBXW7 appears to have a significant impact on promoting EMT. In vivo evidence has demonstrated that the co-inactivation of Fbxw7 and Pten in murine models leads to stepwise progression from endometrioid intraepithelial neoplasia to invasive adenocarcinoma and, ultimately, to carcinosarcoma [23,74]. Notably, all resulting carcinosarcomas exhibited heterologous sarcomatous elements, suggesting that FBXW7 may also contribute to the development of heterologous components [75].

WT1 has been recognized as an independent negative prognostic marker for overall survival, highlighting its biological and clinical significance in uterine sarcomas [13].

Han et al. demonstrated that aurora kinase expression may serve as a novel adverse prognostic biomarker in uterine carcinosarcoma, given its apparent association with lymphatic metastasis, vascular invasion, and omental dissemination. High expression levels of both phospho-aurora kinase A and aurora kinase B have been identified as predictors of reduced progression-free survival (*p* = 0.049). Furthermore, aurora kinase activity appears to promote bidirectional tumor dissemination through both lymphatic and hematogenous pathways. These findings highlight the biological relevance of aurora kinases in UCS pathogenesis and suggest that their inhibitors may represent a promising therapeutic strategy [40].

HER2 oncogene expression in uterine carcinosarcoma has been reported with considerable variability in the literature, ranging from 6% to 56% [41]. The presence of HER2 is a well-established adverse prognostic marker in various malignancies, such as uterine serous carcinoma [42,43,76]. Further research is warranted to elucidate the potential therapeutic benefit of HER2-targeted treatments in uterine carcinosarcoma and their impact on improving prognosis [77,78].

The characteristics and the results of the included studies are summarized in Table 2.

### 3.3. Endometrial Stromal Sarcoma (ESS)

Endometrial stromal sarcoma is a rare subtype of uterine mesenchymal neoplasm representing approximately 1% of all uterine malignancies and less than 10% of uterine sarcomas [44].

#### 3.3.1. Stage, Grade, and Surgical Approach

The ESS classification has changed over the years, having been historically divided into two types: low-grade endometrial stromal sarcoma (LGESS) and undifferentiated endometrial sarcoma (UES) [79]. However, in 2014, the WHO classified endometrial stromal tumors (ESTs) based on their immunohistochemistry and molecular findings into four subtypes: endometrial stromal nodule (ESN), low-grade ESS (LGESS), high-grade ESS (HGESS), and undifferentiated uterine sarcoma (UUS) [80]. This categorization stems from findings indicating that LGESS and HGESS display relatively simple karyotypes at the molecular level, in contrast to UUS, in which specific chromosomal rearrangements are absent [80]. Several studies [45,46,47,48,49,50,81,82,83] have identified tumor size, mitotic count, tumor stage, histologic grade, margin involvement, menopausal status, and age as factors of prognostic importance.

Despite observed differences in clinical outcomes, considerable controversy remains regarding the factors that determine prognosis, with studies reporting that early-stage disease, low mitotic count, and the absence of deep myometrial invasion are associated with improved overall survival, whereas patient age and adjuvant therapy have no significant impact [51,84]. Conversely, other studies—such as that of Nordal et al.—have identified free surgical margins at primary resection as the strongest prognostic factor, followed by tumor grade, tumor size, and menopausal status [45]. Furthermore, Bodner et al. identified early tumor stage, limited myometrial invasion, and low mitotic count as prognostic factors associated with prolonged overall survival in patients with ESS, while age, histologic grade, and the use of adjuvant therapy did not appear to impact overall survival [84]. Ongoing uncertainty about the natural history and prognostic indicators of this disease continues to hinder the development of a standardized management approach.

Surgery, typically involving hysterectomy and bilateral salpingo-oophorectomy (BSO), has consistently been regarded as the most effective treatment for uterine sarcomas [52,85,86]. In line with previous studies, Nordal et al. found that early tumor stage (FIGO Stage 1) is the most significant prognostic factor in ESS [20,45,63]. A low FIGO stage seems to facilitate complete primary surgical resection, thereby improving the likelihood of long-term survival in patients with ESS. However, the prognostic value of lymph node metastasis and the therapeutic role of lymphadenectomy remains controversial [53,87,88].

As such, the prognosis of patients diagnosed at an early stage is generally excellent [50,54]. Nevertheless, late recurrences have been reported [50,89]. Given the above, along with the younger age at diagnosis and favorable early-stage prognosis, the authors of a recent systematic review argued that ovarian preservation should be considered to avoid the adverse effects of surgical menopause [90].

However, data regarding the impact of ovarian preservation on the recurrence of endometrial stromal sarcoma are contradictory. While patients who undergo BSO tend to have improved DFS [54,90], one study showed BSO does not appear to significantly influence time to recurrence or OS [89]. However, that study included heterogeneous patient populations encompassing all disease stages and was limited by a relatively short follow-up period. A Gynecologic Cancer InterGroup (GCIG) consensus review indicated that ovarian preservation does not adversely affect survival. As a result, ovarian preservation is recommended, particularly in younger women, to avoid menopausal symptoms and maintain quality of life [89].

Contrary to the above, a recent retrospective analysis suggested that oophorectomy in patients with retained ovaries is associated with improved DFS compared to patients without oophorectomy [91].

The prognostic relevance of lymph node metastasis and the role of complete lymphadenectomy in endometrial stromal sarcoma remains a subject of ongoing debate [53,55,87,88]. Reported rates of nodal metastasis vary widely, with incidence ranging from 6.6% to 7% and prevalence from 0% to 37% [54,56,92,93]. A retrospective analysis of factors affecting the recurrence of endometrial stromal sarcomas in the European Journal of Obstetrics and Gynecology was unable to confirm a clear prognostic impact of lymphadenectomy. Nevertheless, the surgical removal of metastatic lymph nodes, along with the resection of visible extra-uterine disease, was associated with improved DFS [91].

Cytoreductive surgery has become a well-established component of treatment for advanced endometrial cancer [57,94]. Evidence from a multicenter retrospective study indicates that reducing tumor burden to less than 2 cm is significantly linked to better survival outcomes in patients with HGESS [95]. Leath et al. also demonstrated that thorough staging and cytoreductive surgery lead to improved disease-free survival in both high-grade (HGESS) and low-grade (LGESS) variants. However, the most effective adjuvant treatment approach remains uncertain [86].

In line with the published literature, Leath et al. also confirmed that HGESS carries a worse prognosis than LGESS, underscoring the need to combine cytoreductive surgery with adjuvant treatment in these high-grade cases [95]. However, it is worth noting that most studies on adjuvant therapy for HGESS have been retrospective and often include cases of UUS, based on earlier WHO classifications [58].

#### 3.3.2. Immunohistochemical/Molecular Markers

Recent advancements have demonstrated that immunohistochemistry is valuable not only for distinguishing between different malignant types but also for assessing the prognosis of tumors in the female reproductive system. However, there are still no immunohistochemical markers that are uniquely specific for diagnosing ESS. Studies have demonstrated that ESS may be positive for several markers, including CD10, vimentin, HHF35, desmin, CD34, cytokeratin (CK), CD99, and smooth muscle actin, as well as estrogen and progesterone receptors [55]. CD10—also referred to as the acute lymphoblastic leukemia antigen—is a neutral endopeptidase located on the cell surface that inactivates biologically active peptides and may represent a molecular marker that correlates with the prognosis of ESS patients [55]. Recent findings by Oliva et al. [96] demonstrated that only 10% of endometrial stromal tumors tested positive for CD10, while Agoff et al. [97] and McCluggage et al. [98] reported the absence of CD10 in four out of four and four out of six high-grade ESS cases, respectively. The authors suggested that reduced CD10 expression in high-grade ESS may be associated with the level of tumor differentiation.

In addition, Youn Jin Choy et al. attempted to demonstrate potential genetic features of UUSs, tumors that, by definition, do not harbor any ESS-specific fusions [31]. In their analysis, the ESS cases included demonstrated between 6 and 36 non-silent somatic mutations per genome. However, these did not involve commonly known mutations such as TP53, KRAS, or PIK3CA. Regarding copy number alterations (CNAs), the study revealed that ESSs contain not only gene fusions specific to ESS but also somatic mutations and copy number alterations involving driver genes, suggesting that gene fusions alone may not be sufficient for the full development of ESS, similar to what has been observed in other types of tumors.

In accordance with the above, several studies have identified a number of genes in undifferentiated uterine sarcomas showing both copy number alterations and corresponding changes in gene expression. Specifically, PRKAR1A, CDH1, RB1, and TP53 were downregulated alongside CNA losses, while EZR was upregulated with a CNA gain. The loss of CDH1, a tumor suppressor gene encoding E-cadherin, has been linked to the development of various cancers [99,100,101] and increased invasiveness and tumor progression.

In 2003, the World Health Organization revised the classification of endometrial stromal sarcomas by removing mitotic count as a criterion and emphasizing nuclear atypia and necrosis as key diagnostic features to distinguish them from low-grade endometrial stromal sarcoma, which has a favorable prognosis (over 90% recurrence-free survival), and undifferentiated endometrial sarcoma, which is associated with poor outcomes. Weiwei Feng et al. investigated whether proliferation biomarkers could predict recurrence in WHO 2003-defined ESS-LG cases. In a cohort of 24 invasive ESS cases, a survival analysis was conducted to assess the prognostic value of traditional mitotic counts (mitotic activity index) from H&E-stained sections, along with immunohistochemical markers of proliferation such as Ki-67 and phosphohistone H3 (PPH3). Recurrence occurred in 3 out of 24 patients (12.5%). All three biomarkers—MAI, PPH3, and Ki-67—showed significant prognostic value, with *p*-values of 0.001, 0.002, and 0.03, respectively [102]. With standardized protocols now available for assessing the mitotic activity index and the immunohistochemical proliferation markers Ki-67 and PPH3, a diagnostic model that incorporates these three indicators may provide enhanced diagnostic utility [102]. Ki-67 is expressed in nearly all phases of the cell cycle—G1, S, and G2—making it a broad marker of cell proliferation; in contrast, the PPH3 antigen is expressed almost exclusively in cells during the late G2 phase and throughout the M phase, where mitotic figures are visible. The prognostic significance of these three proliferation markers is particularly noteworthy, as they reflect distinct phases of the cell cycle and exhibit only partial overlap in their expression patterns. These findings demonstrate that the elevated levels of these markers in recurrent ESS-LG cases truly represent a biologically increased growth rate [102].

The cell proliferation index, evaluated using the MIB-1 antibody targeting the Ki-67 antigen, is widely utilized and accepted owing to its clear, high-contrast staining pattern and compatibility with standard laboratory procedures. Numerous studies have demonstrated its diagnostic and prognostic utility. In a small series of 11 low-grade ESS cases, it accurately identified the 2 patients who went on to develop recurrent disease [103]. In summary, the combined evaluation of MAI, Ki-67, and PPH3 may be valuable in cases of low-grade ESS in identifying approximately 10% of patients who are at an elevated risk of recurrence.

Other studies have also demonstrated a link between high recurrence risk and increased cellular proliferation in LGESS [57]. Additionally, ESSs have been shown to express the MIB-1 proliferation marker significantly more frequently than endometrial stromal nodules (ESNs) [104]. The role of EGFR (epidermal growth factor receptor) in ESS remains unclear. While up to 70% of low-grade ESS cases have shown positive EGFR expression [104], suggesting the potential for targeted therapy using monoclonal antibodies against EGFR, other studies have reported much lower expression rates (as low as 11%), with no evidence of EGFR gene amplification. Therefore, findings of EGFR overexpression in the absence of gene amplification should be interpreted with caution [102].

The characteristics and results of the included studies are summarized in Table 3.

### 3.4. Strengths and Limitations

Our study comprises an extensive and detailed review of several clinicopathological recurrence predictors in cases of u-LMS, CS, and ESS. It incorporates the data and results of several study groups spanning a period of more than 35 years, from 1986 to 2024, providing a historical and holistic perspective. However, several limitations should be acknowledged. First, the studies included were mostly retrospective cohorts and case–controls, which are prone to underlying bias. Second, several of the molecular biomarkers analyzed in our review remain under investigation, with their clinical utility and implementation still unclear and preliminary. Furthermore, several studies have provided results based on small sample sizes—a factor that could limit the generalizability of their results. Lastly, it should be underlined that the introduction of the new classification system in uterine cancer has significantly altered the defined stages of carcinosarcoma [64]. This new staging system seems to perform better in terms of predicting disease prognosis, and this advancement should be taken into account when considering the results of studies published prior to its implementation.

### 3.5. Future Directions

Despite recent advancements in our understanding of their genetic and molecular characteristics, high-grade variants such as HG-ESS and UUS present considerable challenges in both diagnosis and treatment given their molecular heterogeneity, aggressive characteristics, and restricted therapeutic alternatives. The necessity for innovative pharmacological approaches is critical, given that traditional treatments—such as chemotherapy and radiation—frequently demonstrate restricted effectiveness. Immunotherapy seems to demonstrate promising outcomes, but additional research is necessary to identify potential responders and the underlying factors associated with increased risk of recurrence or overall poor prognosis. Furthermore, future research should focus on identifying pertinent mutations in diagnostically challenging cases, further improving the accuracy of conventional diagnostics. While surgery is currently the primary treatment modality for uterine sarcomas, studies seem to highlight that the incorporation of molecular typing and molecular-specific individualized therapies may enhance patient outcomes.

## 4. Conclusions

Over the past few years, advancements in the field of next-generation sequencing have enabled the identification of multiple genetic anomalies—particularly fusions—in various uterine mesenchymal tumors. This has led not only to the distinction of histological patterns that further enhance the existing categorization and treatment options for sarcomas but also the identification of women who display an increased risk of recurrence, poor disease-free survival, and an overall adverse prognosis. The stratification of this risk necessitates a deep knowledge of the underlying clinical, histological, and molecular risk factors. This is a crucial component in addressing the most appropriate way to initiate consultation, guide management, and—in view of the novel treatment modalities—individualize the care of those women based on the histological and molecular characteristics of their disease.

## Figures and Tables

**Table 1:** Characteristics and results of the included studies evaluating the clinicopathological predictors of recurrence in uterine leiomyosarcomas. Abbreviations: OS: overall survival; PFS: progression-free survival; DSS: disease-specific survival.

Author	Year	Type of Study	Sample Size (Cases/Controls)	Parameter Assessed	Significant Results
**Wang et al. [6]**	2022	Retrospective cohort	155	Tumor size Tumor stage	Tumor size was an independent prognostic factor for OS and PFS. Tumor stage was significantly associated with PFS.
**Denschlag et al. [7]**	2007	Retrospective cohort	30	Age Tumor stage	Age and advanced stage were significant predictors of OS.
**D’Angelo et al. [8]**	2009	Case–control study	34/18	Tumor stage Ki-67 P53 Bcl-2	Negative/low expression of Ki-67 and p53 and strong expression of bcl-2 were associated with decreased rates of recurrence.
**Kapp et al. [9]**	2008	Retrospective cohort	1396	Age Tumor stage Tumor grade	Age and tumor stage and grade were independent predictors of DSS.
**Tirumani et al. [10]**	2014	Retrospective cohort	113	Age Tumor stage	Age and tumor stage were predictive factors for metastases.
**George et al. [11]**	1986	Retrospective cohort	81	Menopausal status Age	Menopausal status was a strong predictor of OS, while age did not demonstrate a significant correlation with prognosis.
**Wang et al. [12]**	2024	Retrospective cohort	33	Age Menopausal status Tumor stage	Menopausal status and advanced stage were significant risk factors for PFS and OS.
**Coosemans et al. [13]**	2011	Retrospective cohort	24	Age Tumor size Tumor stage Wilms tumor gene 1	Tumor stage and Wilms tumor gene 1 were independent prognostic factors for OS.
**Giuntoli et al. [14]**	2003	Retrospective cohort	208	Tumor stage Tumor grade	Tumor grade and stage were significantly correlated with worse DSS.
**Abeler et al. [15]**	2009	Retrospective cohort	235	Tumor stage Tumor size Mitotic index	Stage of disease, tumor size, and mitotic index were significant prognostic factors in leiomyosarcomas.
**D’Angelo et al. [16]**	2011	Retrospective cohort	84	Tumor size Mitotic index Ki-67 Bcl-2	Increased tumor size (>10cm), high mitotic index, positive ki-67, and negative bcl-2 were associated with poor prognosis.
**Chen et al. [17]**	2023	Retrospective cohort	102	Age Tumor size Mitotic index Ki-67 P53	Increased age, larger tumor size, higher mitotic index, and presence of ki-67 were independent poor prognostic factors.
**Dermawan et al. [18]**	2024	Retrospective cohort	238	Tumor size Mitotic index P53 chr20q amplification/ATRX mutations	Tumor size and mitotic index were associated with inferior PFS and DSS. Concurrent TP53 mutation and chr20q amplification/ATRX mutations were associated with increased risk of recurrence.
**Lusby et al. [19]**	2013	Retrospective cohort	349	Mitotic index B-catenin Bcl-2 Ki-67 EGFR	b-catenin expression was associated with intraperitoneal recurrence. Higher mitotic index was associated with poor prognosis. Increased bcl-2 expression was associated with longer DSS.
**Kildal et al. [20]**	2009	Retrospective cohort	231	B-catenin	b-catenin was associated with poor survival in univariate but not multivariate analyses.
**Vadasz et al. [21]**	2023	Retrospective cohort	34	Dystrophin	Dystrophin positivity tends to have a better OS.

**Table 2:** Characteristics and results of the included studies evaluating the clinicopathological predictors of recurrence in uterine carcinosarcomas. Abbreviations: OS: overall survival; PFS: progression-free survival; DSS: disease-specific survival; RFS: relapse-free survival; BMI: body mass index; EMT: epithelial–mesenchymal transition; FIGO: Federation of Gynecology and Obstetrics; UCS: uterine carcinosarcoma; MMMT: malignant mixed Müllerian tumor; LVSI: lymphovascular space invasion; LN: lymph node; DFI: disease-free interval; DFS: disease-free survival; EC: endometrial carcinoma/cancer; ESMO: European Society of Medical Oncology; USPC: uterine serous papillary endometrial carcinoma; ECOG: Eastern Cooperative Oncology Group.

Author	Year	Type of Study	Sample Size (Cases/Controls)	Parameter Assessed	Significant Results
**Gotoh et al. [22]**	2019	Retrospective cross-sectional study	109	Molecular subtypes	Four genomic subtypes (MSI, POLE, CNH, and CNL) are significantly associated with PFS and OS. Higher age for the CNH subtype. Lower BMI for the POLE subtype. Across 40 SNV/indel driver genes, 9 and 22 (55%) genes were identified as significantly correlated with better OS and PFS, respectively. EMT score was not significantly correlated with histological grade and FIGO stage or RFS and OS.
**Cherniack et al. [23]**	2017	Retrospective cohort study	57	Genomic analysis Proteomic analysis	In total, 73% of all mutations and 82% of mutations in significantly mutated genes were clonal. No significant association was found between EMT score and clinical outcomes.
**Zhao et al. [24]**	2016	Retrospective cohort study	68	Mutational landscape	Carcinomatous and sarcomatous elements derive from a common precursor with mutations typical of carcinomas. Significantly increased frequency of somatic mutations in histone genes (H2A, H2B); amplifications of the histone gene locus on chr6p; and significant amplification of TERT 5p, PIK3CA, and MYC and the TP53 and MBD3 genes.
**Nordal et al. [25]**	1997	Retrospective cohort study	46	Age Histological type Tumor size Tumor stage Tumor grade Parity	Age, tumor stage, and the presence of serous or clear-cell components were independent predictors of UCS.
**Matsuo et al. [26]**	2018	Retrospective cohort study	11.000	Age Race Marital status Registry area Histology type Tumor stage	Older age, black race, Eastern U.S. residence, and divorced/widowed marital status are predictors for increased risk of UCS compared with other histology types. UCS is more associated with distant metastases than other histology types.
**Sherman et al. [27]**	2003	Retrospective cohort study	20.192	Race Tumor stage Tumor grade Age	Black race is associated with significantly higher incidence of UCS and worse survival/mortality.
**Zelmanowicz et al. [28]**	1998	Prospective case–control	453 cases, 320 controls	Age Race Educational status BMI Parity Smoking Estrogen use Oral contraceptives	UCS (MMMT) has an increased risk of incidence in African American women. Obesity, long-term or recent exogenous estrogen use, and nulliparity are linked to an increased risk of endometrial cancer and UCS. Oral contraceptive use and cigarette smoking were associated with a decreased risk of developing EC and MMMT.
**Abdulfatah et al. [29]**	2019	Retrospective cohort study	196	Age Race Adjuvant treatment Recurrence/site of recurrence Histologic subtype Gross appearance Tumor size Tumor stage (FIGO) Tumor grade Depth of myometrial invasion LVSI LN metastasis Pelvic wash cytology	Tumor size, myometrial invasion, LVSI, LN metastasis, advanced stage (stages III–IV), sarcomatous component on recurrence, sarcoma dominance, and positive cytology were significantly associated with shorter DFI. Serous histology and rhabdomyoblastic differentiation were significantly associated with worse 3 yr OS. Age at diagnosis, race, recurrence site, adjuvant treatment, pattern of myometrial invasion and collision, and tumor necrosis did not significantly impact DFI.
**Matsuo et al. [30]**	2016	Retrospective cohort study	906	Age Ethnicity BMI Pregnancy history Personal malignancy history Preoperative CA-125 Prior tamoxifen use History of pelvic irradiation Tumor size Tumor stage Lymphovascular space invasion (LVSI) Depth of myometrial tumor invasion Histology of metastatic sites Tumor grade Postoperative chemotherapy Postoperative radiotherapy	High-grade/heterologous and high-grade/homologous histology patterns of UCS, older age, residual disease at surgery, large tumor, sarcoma dominance, deep myometrial invasion, LVSI, and advanced-stage disease are independently associated with decreased PFS. Postoperative chemotherapy and radiotherapy were significantly associated with improved PFS, but only postoperative chemotherapy is an independent predictor of PFS. Carcinoma components tended to spread lymphatically, while sarcoma components tended to spread loco-regionally.
**Harano et al. [31]**	2016	Retrospective cohort study	486	Age Tumor stage Performance status Pregnancy history Menopausal status Tumor markers (CA-125, CA19-9) Histological type Myometrial invasion LVSI Invasion in the parametrium Ovarian metastasis Cytology of the pelvic washing Adjuvant therapy Type of surgery	Stage III–IV disease, CA-125 level, and LVSI were significantly associated with shorter median DFS. Stage III–IV disease, performance status 2–4, ≥50% myometrial invasion depth, and postsurgical residual tumor size >1 cm were significantly associated with shorter median OS. Pelvic lymph node lymphadenectomy was associated with improved DFS and OS.
**de Jong et al. [32]**	2011	Retrospective cohort study	40	Age Tumor stage Tumor type Tumor grade Myometrial invasion Vascular invasion Recurrence Peritoneal washing Metastatic sites Molecular markers	The epithelial component caused the majority of metastases and vascular invasion. Patients with a non-endometrioid epithelial component had worse survival.
**Kim et al. [33]**	2023	Meta-analysis	1.594	Histological type	Gynecologic carcinosarcoma is histologically a biphasic tumor that comprises epithelial and mesenchymal components. Heterologous component in gynecologic carcinosarcoma is an independent prognostic factor for OS.
**Network CGAR et al. [34]**	2013	Retrospective cross-sectional study	373	Genomic analysis Transcriptomic analysis Proteomic analysis	ECs are classified into four categories: POLE ultramutated, microsatellite instability hypermutated, copy-number low, and copy-number high. The genomic features of endometrial carcinomas permit a reclassification that may affect post-surgical adjuvant treatment for women with aggressive tumors.
**Talhouk et al. [35]**	2015	Retrospective cohort study	152	Age BMI Tumor stage Tumor grade Histology type LVSI Lymph node metastases Adjuvant treatment	Molecular classification of ECs can be achieved using clinically applicable methods on formalin-fixed paraffin-embedded samples and provide independent prognostic information beyond established risk factors.
**Talhouk et al. [36]**	2017	Retrospective cohort study	319	Age BMI Tumor stage Tumor grade Histology type LVSI Myometrial invasion Lymph node metastases Adjuvant treatment	Tumors with POLE mutations had the most favorable prognosis, and those with p53abn had the worst prognosis. There were no significant differences in survival between the ESMO low-risk and intermediate-risk groups.
**Kommoss et al. [37]**	2018	Retrospective cohort study	452	Age BMI Tumor stage Tumor grade Histology type LVSI Myometrial invasion Lymph node metastases Adjuvant treatment Molecular features	About 10% of ECs harbored POLE exonuclease domain mutations, associated with excellent outcomes. Very poor outcomes observed in women with p53abn tumors. Patients with MMR-D tumors (28%) exhibited worse OS, DSS, and PFS when compared with p53wt. Women with ECs harboring POLE EDMs were younger than those with other subtypes and thinner than women with MMR-D and p53wt ECs. Lower proportion of grade 3 endometrioid carcinomas within the POLE subgroup.
**Britton et al. [38]**	2019	Retrospective cohort study	257	Age BMI FIGO stage Tumor grade Histological subtype LVSI Myometrial invasion Lymph node metastases Radiation Chemotherapy Post-surgical treatment Progesterone therapy Clinical phenotype Ethnicity Parity Molecular features	Women with p53wt ECs had younger age and higher BMI. MMRd and p53abn tumors were more likely to be advanced stage (III/IV), high-risk (ESMO), and receive chemotherapy.
**Castilla et al. [39]**	2011	Retrospective cross-sectional study	96	Genomic analysis Proteomic analysis	Interplay between E-cadherin repressors and miRNAs links EMT activation with maintenance of stemness in ECs.
**Han et al. [40]**	2017	Retrospective cohort study	24	Age BMI FIGO stage Menopausal status Adjuvant therapy Myometrial invasion LVSI LN metastasis Adnexal/omentum involvement	Phospho-aurora kinase A and aurora kinase B showed significantly higher expression in the carcinomatous component. High expression of phospho-aurora kinase A was associated with lymphatic metastasis, such as positive pelvic lymph nodes and omental involvement. Overexpression of aurora kinase B was related to vascular invasion. High expression of both phospho-aurora kinase A and aurora kinase B was a prognostic factor for PFS in UCS.
**Rottmann et al. [41]**	2020	Retrospective observational study	80	HER2 protein	All HER2-positive carcinosarcomas had either a serous or a mixed carcinoma component.
**Santin et al. [42]**	2005	Retrospective cohort study	30	HER-2/neu gene	HER-2/neu gene amplification in USPC was found to be an important prognostic indicator for poor outcomes that occur more frequently in African American patients than in Caucasian patients.
**Erickson et al. [43]**	2020	Retrospective cohort study	169	Age BMI Race/ethnicity ECOG performance status Serous component Depth of invasion Tumor size Involvement of a polyp LVSI Surgical staging Adjuvant treatment Recurrence Survival	Significantly more recurrences in the HER2-positive cohort. HER2-positive tumors were associated with worse PFS and OS. HER2 positivity appears to be a prognostic biomarker in women with stage I uterine serous carcinoma.

**Table 3:** Characteristics and results of the included studies evaluating the clinicopathological predictors of recurrence in endometrial stromal sarcomas. Abbreviations: ESS: endometrial stromal sarcoma; MMP: matrix metalloproteinase; ER: estrogen receptor; PR: progesterone receptor.

Author	Year	Type of Study	Sample Size	Parameter Assessed	Significant Results
**JK Chan et al. [44]**	2022	Retrospective cohort	831	Tumor grade Tumor stage Race Age	The survival rate in patients with grade 1 and 2 disease was larger than in those with grade 3 disease. Older age, Black race, advanced stage, higher grade, lack of primary surgery, and nodal metastasis were independent prognostic factors for poorer survival.
**R R Nordal et al. [45]**	1996	Retrospective cohort	48	Cellular atypia Tumor diameter Tumor stage Menopausal status Primary surgery	Free resection margins at primary surgery, malignancy grade, tumor diameter, and menopausal status are important prognostic factors in endometrial stromal sarcoma.
**Burhuck et al. [46]**	1989	Case–control study	31	Endolymphatic stromal myosis Primary surgery Adjuvant radiotherapy	There is a relationship between endolymphatic stromal myosis, recurrence rate of ESS, and the type of therapy.
**Janine et al. [47]**	1990	Case report	15	Tumor stage Adjuvant radiotherapy	After surgical resection, patients with low-grade tumors probably do not require treatment until relapse, whereas adjuvant radiotherapy should be given to patients with high-grade tumors.
**G P Sutton et al. [48]**	1986	Retrospective cohort	43	Estrogen receptors of ESS Progesterone receptors	Patients with estrogen-receptor-positive sarcomas were more likely to survive longer than one year than those with estrogen receptor-negative tumors.
**Liokumovich et al. [49]**	2015	Retrospective cohort	11	Matrix metalloproteinases Extracellular matrix invasion Extracellular matrix degradation Staining Tumor grade	MMP expression does not appear to predict disease outcomes in endometrial stromal sarcoma.
**Karen et al. [50]**	1990	Retrospective cohort	117	Tumor stage Mitotic index Cytologic atypia	Mitotic index and cytologic atypia are not predictive of tumor recurrence for patients with stage I tumors.
**Bodner et al. [51]**	2001	Retrospective cohort	31	Tumor grade Mitotic count Myometrial invasion	Association between early tumor stage, low myometrial invasion, and low mitotic count with a lengthened overall survival in patients with ESS.
**Ashraf-Ganjoei et al. [52]**	2011	Case–control study	14	Tumor grade Low-grade ESS Myometrial invasion Mitotic count Disease-free survival	Patients with no myometrial invasion and low mitotic count have longer disease-free survival.
**C Chu et al. [53]**	2003	Retrospective cohort	22	Tumor grade Estrogen receptor type a Estrogen receptor type b Progestin receptors Exposure to hormones Prognosis of the disease Reverse-transcription polymerase chain reaction (RT-PCR)	Estrogen replacement therapy might be harmful for patients with low-grade endometrial stromal sarcoma. The absence of ERβ expression in endometrial stromal sarcomas, as opposed to its presence in normal endometrial stromal cells, indicates that the loss of ERβ may serve as a marker of malignancy. Progestin therapy ought to be regularly considered as part of adjuvant treatment and for managing recurrent endometrial stromal sarcomas.
**Yoon et al. [54]**	2014	Retrospective cohort	114	Tumor stage Estrogen receptors Progesterone receptors Bilateral salpigo-oophorectomy Cytoreductive resection of lesions	Stage, ER/PR expression, and lymph node metastasis are strongly linked to overall survival in endometrial stromal sarcoma. Bilateral salpingo-oophorectomy is considered the primary treatment. Surgical removal of recurrent lesions (cytoreductive resection) should be considered to enhance survival outcomes in patients with endometrial stromal sarcoma.
**Li He et al. [55]**	2014	Retrospective cohort	72	CD 10 Vimentin Tumor grade Total hysterectomy with bilateral salpingo-oophorectomy Total hysterectomy with bilateral salpingo-oophorectomy	The absence of CD10 expression in high-grade ESS suggests that reduced CD10 levels may be associated with tumor differentiation. CD10 may serve as a molecular marker associated with the prognosis of patients with endometrial stromal sarcoma. CD10-positive patients showed a longer DFS. Total hysterectomy with bilateral salpingo-oophorectomy, followed by total hysterectomy with bilateral salpingo-oophorectomy, is likely to lead to improved treatment outcomes.
**Bai et al. [56]**	2014	Retrospective cohort	153	Hysterectomy with bilateral BSO Ovary-sparing treatment	**Hysterectomy with bilateral salpingo-oophorectomy and complete resection of visible lesions should be considered the standard treatment for low-grade endometrial stromal sarcoma.** **Ovary-sparing procedures may be an option for younger women without cervical involvement.** **Myomectomy should be reserved only for young patients with a strong desire to preserve fertility.**
**Alagkiozidis et al. [57]**	2015	Retrospective cohort	168	Cytoreduction Gross residual disease Tumor stage	Optimal debulking has been linked to better survival outcomes in various types of cancer. There is no correlation between histologic type and the ability to achieve optimal debulking.
**Alagkiozidis et al. [57]**	2015	Retrospective cohort	168	Cytoreduction Gross residual disease Tumor stage	Optimal debulking has been linked to better survival outcomes in various types of cancer. There is no correlation between histologic type and the ability to achieve optimal debulking.
**Dahhan et al. [58]**	2009	Retrospective study	13	Hysterectomy Bilateral oophorectomy Hormonal therapy Residual tumor Recurrent disease	Hormonal therapy demonstrates a high response rate in cases of measurable residual or recurrent low-grade endometrial stromal sarcoma and should be regarded as the preferred treatment option for patients whose recurrent disease is not amenable to surgical resection.

## Data Availability

No new data were created or analyzed in this study.

## Abbreviations

The following abbreviations are used in this manuscript:

u-LMS	uterine leiomyosarcoma
CS	carcinosarcoma
ESS	endometrial stromal sarcoma
OS	overall survival
DFS	disease-free survival
MF	mitotic figures
HPF	high-power field
WT1	Wilms tumor gene 1
APC	annual percent change
PFS	progression-free survival
LVSI	lymphovascular space invasion
TCGA	The Cancer Genome Atlas
POLEmut	POLE-ultra-mutated
MSI/MMRd	microsatellite instability/mismatch repair-deficient
NSMP	no specific molecular profile
TP53mut/p53abn	TP53-mutant/p53-abnormal
EMT	epithelial–mesenchymal transition
LGESS	low-grade endometrial stromal sarcoma
UES	undifferentiated endometrial sarcoma
BSO	bilateral salpingo-oophorectomy
GCIG	Gynecologic Cancer InterGroup
CNA	copy number alterations
PPH3	Phosphohistone H3
EGFR	epidermal growth factor receptor
